# A League-Wide Evaluation of Factors Influencing Match Activity Profile in Elite Australian Football

**DOI:** 10.3389/fspor.2020.579264

**Published:** 2020-11-06

**Authors:** Alireza Esmaeili, Patrick Clifton, Robert J. Aughey

**Affiliations:** ^1^Institute for Health and Sport (iHeS), Victoria University, Melbourne, VIC, Australia; ^2^Australian Football League (AFL), Melbourne, VIC, Australia

**Keywords:** external load, speed, physical performance, AFL, player characteristics, environmental conditions, game variables

## Abstract

**Introduction:** Accurate interpretation of activity profile data requires an understanding of the variables influencing player movement during matches.

**Methods:** Over 65,000 stints (player rotations) from all 207 matches of the 2018 Australian Football League (AFL) season were evaluated. The relative activity profile including total distance per minute (TD), high-speed running distance per minute (HSR) and Player Load^TM^ per minute (PL) was determined for each stint and analysed against a range of match-related, player-related and environment-related predictor variables using multivariate linear mixed modelling. Effect size statistics along with the uncertainty in the estimates (95% confidence interval) were used to interpret the findings.

**Results:** The smallest important effects on TD, HSR, and PL were calculated as 1.5%, 5.5%, and 2.4%, respectively. Stint duration had small to moderate negative effects on TD (−6%), PL (−7.7%), and HSR (−13%), while recovery duration between stints had a small positive effect on HSR (+7%). There were moderate reductions in TD (−8%), HSR (−23%), and PL (−9.6%) in the last quarter compared to the first quarter of matches, while similar reductions existed in subsequent stints compared to the first stint in each quarter. Moderate to large differences of up to 9% in TD, 48% in HSR and 12% in PL existed between positions. The TD of less experienced players was slightly higher than their more experienced counterparts (2–3%). A 5% increase in body mass was associated with a small reduction in HSR (−5.5%). There were small reductions in TD (−2%), HSR (−10%), and PL (−3%) during the Finals Series compared to the Premiership Season. Moderate levels of rainfall during matches and higher apparent temperatures had small negative effects on TD (−2%) and HSR (−6 and −9%). The number of days break between matches, score margin, match outcome, ground hardness, ground size, and traveling for the current or the previous match had trivial effects on the activity profile.

**Conclusion:** Player position and stage of the match (quarter) had the largest effects on match activity profile while stint duration, recovery duration, stint timing, professional experience, body mass, stage of the season, and weather conditions also had substantial effects.

## Introduction

Match activity profile data are increasingly being used by sport governing bodies, coaches, fans, media, and researchers to gain a greater understanding of the game (Aughey, 2011a). Understanding the effects of match-related, player-related, and environment-related variables on player physical performance will contribute toward a more accurate interpretation of match activity profile data and improved data-assisted decision making.

The vast majority of publications in this area have used Global Positioning System (GPS) data from a single club to evaluate the effects of a limited number of variables on match activity profile (Mooney M. et al., 2013; Murray et al., 2014; Sullivan et al., 2014; Kempton and Coutts, 2016; Ryan et al., 2017, 2018; Dillon et al., 2018). These studies have contributed toward the identification of variables affecting player movement and advanced our understanding of match physical performance metrics.

In recent years a centralised approach toward the collection and sharing of match activity profile data has been adopted across the Australian Football League (AFL), where all clubs use athlete tracking devices from the same manufacturer (Catapult) and make the match data available to the AFL's official match statistics data supplier (Champion Data, Melbourne, Australia). Availability of such a large league-wide dataset combined with the use of mixed modelling provides the opportunity to simultaneously evaluate a larger subset of potentially influential variables, which will in turn allow for estimation of the pure effect of each variable after adjusting for the effects of all other variables in the model. The concept of cooperation among competitors and the associated benefits have recently been highlighted in the literature (Ramírez-López et al., 2020). The aim of this study was to evaluate the effects of a range of match-related, player-related, and environment-related variables on match activity profile across an entire season of the Australian Football League in order to further our understanding of the sport.

## Methods

### Data Collection

Activity profile data of over 65,000 stints from all 207 matches of the 2018 Australian Football League (AFL) season were obtained from Champion Data. A total of 657 players (mean age ± SD; 24.4 ± 3.7 years) from 18 clubs contributed to the analysed observations. The study conformed to the Declaration of Helsinki and protocols were approved by the Victoria University Human Research Ethics Committee.

The activity profile of all 44 players involved in each match was recorded using the Catapult S5 GPS (10 Hz)/accelerometer (100 Hz) units (firmware versions 7.36, 7.38, 7.40, and 7.42; Catapult Sports, Melbourne, Australia). These units were replaced by Catapult T6 Local Positioning System (LPS) units (firmware version 5.04) for matches played at the roofed Marvel Stadium. The raw tracking data were processed using the OpenField software (versions 1.17 and 1.18). The updates to firmware and software versions throughout the season did not contain any changes that would have affected the variables of interest.

Half-hourly weather data recorded at the closest weather station to each match venue in each city were obtained from the Australian Bureau of Meteorology for the entire length of the season. Apparent temperature (a function of ambient temperature, humidity and wind speed) (Steadman, 1994) was determined at the start time of each match along with the amount of rainfall for the ~2.5 h duration of the match. Rainfall and wind speed were set to zero for matches played at the roofed Marvel Stadium.

Ground hardness was measured typically 1–3 days prior to each match using a Clegg Impact Soil Tester with the 2.25 kg hammer and a drop height of 455 mm. The mean of first drops at 45 measurement points across the ground was used for the analysis.

Travel distance for players from each club in each round was calculated in the Statistical Analysis System (SAS) from the data on match location and the clubs' home city using the GEOCODE procedure and GEODIST function. Travel distance in the previous round was set to zero for players who had not played an AFL match in the previous round.

Professional experience was defined as the number of years a player was included in the AFL list of a club, inclusive of the current season and categorised into three levels (1–2, 3–6, and 7+ years) to allow for evaluation of possible non-linear effects of experience. A single body mass measurement taken by Club staff at later stages of preseason was provided to the AFL.

Player position was determined by Champion Data at the end of the season using a proprietary algorithm based on the location of players on the ground relative to the ball at every stoppage along with feedback from AFL Club staff. Positions were classified into one of four groups: key defender, key forward, ruck and nomadic.

### Statistical Analysis

The relative activity profile including total distance per minute (average speed), high-speed running (>5 m.s^−1^, >18 km.h^−1^) distance per minute and Player Load^TM^ per minute was determined for each stint and analysed against a range of match-related, player-related, and environment-related predictor variables using multivariate linear mixed modelling. Data were analysed in Statistical Analysis System (version 9.4, SAS Institute, Cary, NC) using the PROC MIXED procedure. Observations with stint duration shorter than 1 min or unrealistic physical outputs (likely due to poor reception or unit malfunction) guided by the distribution of observations were excluded from the analysis (4% of observations in total). The dependent variables (relative activity profile metrics) were log-transformed to deal with the non-uniformity of effects and error, and allow for the effects to be expressed as percentages (Hopkins et al., 2009).

The fixed effects in the model were stint duration, recovery duration (between-stint bench time within each quarter), stint timing (first vs. subsequent stint in each quarter), quarter, stage of the season (Premiership Season vs. Finals Series), days break (≤6 vs. ≥7), travel (>100 km) for the current and the previous round (yes/no), match outcome (win vs. loss), score margin at the end of the match, position, professional experience level, body mass, rainfall during the match (dry, light ≤1 mm, moderate >1 mm), apparent temperature, ground hardness, and ground size. The random effects were player identity, club identity, and match identity to account for the repeated measure nature of the observations within each cluster. The interaction of player identity and stint duration was also included to account for the individual differences in the effects of stint duration. In the absence of any concurrent validation studies of the Catapult S5 and T6 models, the type of tracking system (GPS vs. LPS) was added to a separate model as a fixed effect to evaluate any possible systematic differences in outputs between the two tracking systems. Match outcome and score margin were entered into the model separately due to the correlations between them. Days break was only included in a separate model as players who did not play an AFL match in the previous round were excluded from this analysis. The plots of the residuals vs. the predicted values as well as the residuals vs. the predictors were evaluated for uniformity to ensure of the appropriateness of linear modelling (Hopkins et al., 2009).

A two standard deviation (SD) increase in the predictor, representing the difference between a typically high (mean + 1SD), and a typically low (mean − 1SD) value, was used to evaluate the effects of continuous variables (Hopkins et al., 2009). The estimates along with their uncertainty (95% confidence interval) were standardised to interpret the findings. The smallest important effects (change in the relative activity profile metrics) were calculated as 0.2 of the between-player standard deviation in these measures within each position (Hopkins et al., 2009). The scale for qualitative effect magnitudes relative to the smallest important effect is as follows: <1x, trivial; 1x to <3x, small; 3x to <6x, moderate, 6x to <10x, large; ≥10x, very large (Hopkins et al., 2009). An effect was considered unclear if the lower and upper confidence limits were both greater than the smallest important effect in opposite directions (Hopkins et al., 2009).

## Results

### Descriptive Statistics and the Smallest Important Effects

The descriptive statistics of all the continuous dependant and independent (predictor) variables in the model are provided in Table 1. Table 2 summarises the between-player differences and the derived smallest important effects (increase or decrease) for each of the relative activity profile metrics.

**Table 1:** Descriptive statistics of the continuous variables.

**Variable**	**Mean ± SD**	**Variable**	**Mean ± SD**
**Total distance** (metres per minute)	131 ± 17^#^	**Body mass** (kg)	87 ± 8.2
**High-speed running distance** [>5 m.s^−1^] (metres per minute)	18.8 ± 9.1^#^	**Apparent temperature** (°C)	14.6 ± 6.0
**Player load**^**TM**^ (arbitrary units per minute)	12.8 ± 2.3^#^	**Ground hardness** (gravities)	59 ± 8.5
**Stint duration** (minutes)	14.0 ± 7.1	**Ground size** (m^2^)	66700 ± 3300
**Recovery duration** (minutes)	4.1 ± 2.0	**Score margin** (points)	33 ± 25

# *Between-player SD of within-player means regardless of position*.

**Table 2:** The between-player differences in activity profile in a typical match and the smallest important effects.

**Position (n)**	**Between-player differences (SD) in a typical match**		
	**Total distance**	**High-speed running**	**Player Load^**TM**^**
Key defender (81)	7.2%	25.7%	10.6%
Key forward (67)	9.2%	31.7%	13.6%
Nomadic (472)	7.3%	27.4%	12.0%
Ruck (37)	4.9%	34.1%	11.2%
Weighted average	7.5%	27.6%	12.0%
Smallest important effect (0.2 of the SD)	1.5%	5.5%	2.4%

The differences between the outputs from the two tracking systems (GPS-LPS) after adjusting for all other variables in the model were trivial. The estimated differences for relative total distance (−0.2%, 95% confidence interval −1.1% to +0.8%), high-speed running (+2.9%, −0.2% to +5.9%) and Player Load™ (−2.3%, −3.5% to −1.3%) provide an indication of a good concurrent validity of the Catapult S5 and T6 models.

### Stint Duration and Recovery Duration

The effects of stint duration and recovery duration are summarised in Table 3. A 14-min increase in stint duration representing the difference between a typically long (mean + 1SD, 21 min) and a typically short (mean − 1SD, 7 min) stint, was associated with small to moderate reductions in relative activity profile metrics. The effects of a 1-min increase in stint duration are also provided for contextual purposes, and represent the typical rate of decline in physical output throughout a stint. Recovery duration (between-stint bench time within each quarter) had a small positive impact on relative high-speed running and trivial effects on relative total distance and Player Load^TM^.

**Table 3:** The effects of stint duration and recovery duration (between-stints bench time).

**Variable**	**Change**	**Effect (95% CI)**		
		**Total distance**	**High-speed running**	**Player load^**TM**^**
Stint duration	+14 min^#^ (+2SD)	−6.4%^**^ **(−6.6 to −6.2)**	−12.4%^*^ **(−13.4 to −11.4)**	−7.7%^**^ **(−7.8 to −7.6)**
Stint duration	+1 min	−0.46% (−0.47 to −0.44)	−0.89% (−0.96 to −0.81)	−0.55% (−0.56 to −0.54)
Recovery duration	+4 min^#^ (+2SD)	+1.0% (0.7 to 1.2)	+6.7%^*^ **(5.6 to 7.9)**	+1.2% (0.9 to 1.5)

*Substantial effects are in bold; Effect size*:

* *small*;

** *moderate*.

# *Representing the difference between a typically short and a typically long stint/recovery duration*.

Separate analysis of the effects of stint duration and recovery duration for players of each position and also experience level (1–2, 3–6, 7+ years) revealed that the rate of decline during a stint and recovery on the bench were not substantially affected by position or professional experience (differences of <1% for total distance, <2% for high-speed running and <1.1% for Player Load^TM^). The only exception was the effect of recovery duration on relative high-speed running where rucks benefited the most (+14.5%, 95% confidence interval +6.8% to +23%) and key defenders did not benefit (−0.8%, −4.9% to +3.4%) from a 4-min recovery between stints. The effects for nomadics and key forwards were close to the mean (+6.8% and +7.2%).

### Quarters and Stint Timing

The effects of quarters and stint timing are presented in Table 4. A consistent decline in physical output throughout the match with a relatively stable decline rate can be observed leading to small differences in activity profile between consecutive quarters and moderate differences between the fourth and the first quarter. The substantial effects of the stint timing are also evident, where the activity profile in subsequent stints (second or higher stint) within each quarter was substantially lower compared to the first stint in each quarter (small to moderate effects).

**Table 4:** The effects of quarters and stint timing.

**Variable**	**Compared to**	**Effect (95% CI)**		
		**Total distance**	**High-speed running**	**Player load^**TM**^**
Q2	Q1	−3.1%^*^ **(−3.3 to −2.9)**	−9.3%^*^ **(−10.2 to −8.4)**	−3.9%^*^ **(−4.2 to −3.7)**
Q3	Q2	−3.1%^*^ **(−3.3 to −2.9)**	−6.7%^*^ **(−7.6 to −5.8)**	−3.4%^*^ **(−3.7 to −3.2)**
Q4	Q3	−2.1%^*^ **(−2.3 to −1.9)**	−8.8%^*^ **(−9.7 to −7.9)**	−2.5%^*^ **(−2.8 to −2.3)**
Q4	Q1	−8.0%^**^ **(−8.2 to −7.8)**	−22.8%^**^ **(−23.5 to −22.0)**	−9.6%^**^ **(−9.8 to −9.4)**
Subsequent stint (in each quarter)	First stint (in each quarter)	−5.4%^**^ **(−5.6 to −5.2)**	−20.5%^**^ **(−21.3 to −19.8)**	−6.6%^*^ **(−6.8 to −6.4)**

*Substantial effects are in bold; Effect size*:

* *small*;

** *moderate*.

### Travel, Days Break, Finals, Match Outcome, and Score Margin

The physical output was lower (small effects) during the Finals Series (play-off phase) compared to the Premiership Season (home and away phase). Travelling for the current match, travelling in the previous round, the number of days break between matches, match outcome and the final score margin did not have any substantial effects on the match activity profile (Table 5). The distribution of days break as a proportion of analysed observations was as follows: <6-d, 1%; 6-d, 27%; 7-d, 45%; 8-d, 21%; 9-d, 4%; >9-d, 1%.

**Table 5:** The effects of travel, days break, finals, match outcome, and score margin.

**Variable**	**Compared to**	**Effect (95% CI)**		
		**Total distance**	**High-speed running**	**Player load^**TM**^**
Travel (>100 km)	No travel (<100 km)	−0.1% (−0.4 to 0.1)	−1% (−2 to −0.02)	+0.5% (0.2–0.7)
Travel previous round	No travel previous round	0% (−0.2 to 0.2)	−0.3% (−1.3 to 0.8)	+0.1% (−0.1 to 0.4)
Days break ≤ 6	Days break ≥ 7	+0.4% (0.2 to 0.6)	+0.7% (−0.1 to 1.4)	+0.7% (0.6 to 0.9)
Finals	Premiership season	−1.7%^*^ **(−3.3 to 0)**	−9.9%^*^ **(−14.8 to −4.8)**	−2.5%^*^ **(−4.6 to −0.4)**
Match outcome Win	Match outcome Loss	+0.5% (0.3 to 0.7)	+1.7% (0.9 to 2.5)	+0.7% (0.5 to 0.9)
Score margin	Change of +50 points (+2SD)	+0.4% (0.2 to 0.5)	+1.4% (0.8 to 2.0)	+0.4% (0.3 to 0.6)

*Substantial effects are in bold; Effect size*:

* *small*.

### Position, Experience, and Body Mass

The effects of position, experience, and body mass are summarised in Table 6. Position had substantial moderate to large effects on the relative match activity profile. All the activity profile metrics were lower for key positions compared to the nomadics. While key defenders had the lowest relative total distance among all positions, rucks had the lowest relative high-speed running, which was nearly half the output by nomadic players.

**Table 6:** The effects of position, experience, and body mass.

**Variable**	**Compared to**	**Effect (95% CI)**		
		**Total distance**	**High-speed running**	**Player load^**TM**^**
Key defender	Nomadic	−9.1%^***^ **(−10.5 to −7.6)**	−20.1%^**^ **(−26 to −15.6)**	−12.1%^**^ **(−14.6 to −9.6)**
Key forward	Nomadic	−5.8%^**^ **(−7.5 to −4.1)**	−15.6%^**^ **(−21.6 to −9.2)**	−9.7%^**^ **(−12.5 to −6.8)**
Ruck	Nomadic	−4.6%^**^ **(−6.9 to −2.3)**	−47.9%^***^ **(−53 to −42)**	−4.8%^*^ ** (−8.8 to −0.6)**
Experience: (1–2 years)	(3–6 years)	+2.8%^*^ ** (1.5 to 4.0)**	+2.3% (−2.7 to 7.4)	+4.5%^*^ ** (2.2 to 6.8)**
Experience: (3–6 years)	(7+ years)	+2.2%^*^ ** (1.2 to 3.2)**	+1.7% (−2.3 to 5.6)	+0.9% (−0.8 to 2.7)
Body mass	Change of +5%	–0.5% (−0.8 to −0.1)	−5.5%^*^ ** (−6.8 to −4.0)**	−1.3% (−1.9 to −0.7)

*Substantial effects are in bold; Effect size*:

* *small*;

** *moderate*;

*** *large*.

The relative total distance of less experienced players was slightly higher than their more experienced counterparts (small effect). However, there were no substantial differences in relative high-speed running between players of different experience level. Higher body mass had a small negative effect on relative high-speed running and trivial effects on relative total distance and Player Load^TM^.

### Weather and Ground Conditions

The effects of weather and ground conditions are presented in Table 7. Moderate levels of rainfall during matches (>1 mm) had small negative effects on relative total distance and high-speed running, while the effects for light rain were trivial. Higher apparent temperatures also negatively affected the match activity profile whereas the effects of ground hardness and ground size were trivial. The number of observations for rainfall levels were as follows: dry, 53895 stints from 181 matches; light rain, 7406 stints from 24 matches; moderate rain, 624 stints from 2 matches.

**Table 7:** The effects of weather and ground conditions.

**Variable**	**Change**	**Effect (95% CI)**		
		**Total distance**	**High-speed running**	**Player load^**TM**^**
Light rain (≤1 mm)	Compared to dry	+0.3% (−0.7 to 1.4)	−2.7% (−6.1 to 0.8)	+0.2% (−1.1 to 1.6)
Moderate rain (>1 mm)	Compared to dry	−2.2%^*^ (−5.6 to 1.3)	−9.2%^*^ (−19 to 1.9)	−2.2% (−6.6 to 2.2)
Apparent temperature	+12°*C* (+2SD)	−2.0%^*^ (−2.6 to −1.3)	−6.1%^*^ (−8.3 to −3.9)	−1.9% (−2.7 to −1.0)
Ground hardness	+17 gravities (+2SD)	+0.5% (−0.3 to 1.3)	+4.1% (1.4 to 6.9)	+1.0% (0 to 2.0)
Ground size	+6,600 m^2^ (+2SD)	+0.9% (0 to 1.7)	+2.8% (0 to 5.7)	−0.3% (−1.3 to 0.8)

*Substantial effects are in bold; Effect size*:

* *small*.

**Figure 1:**
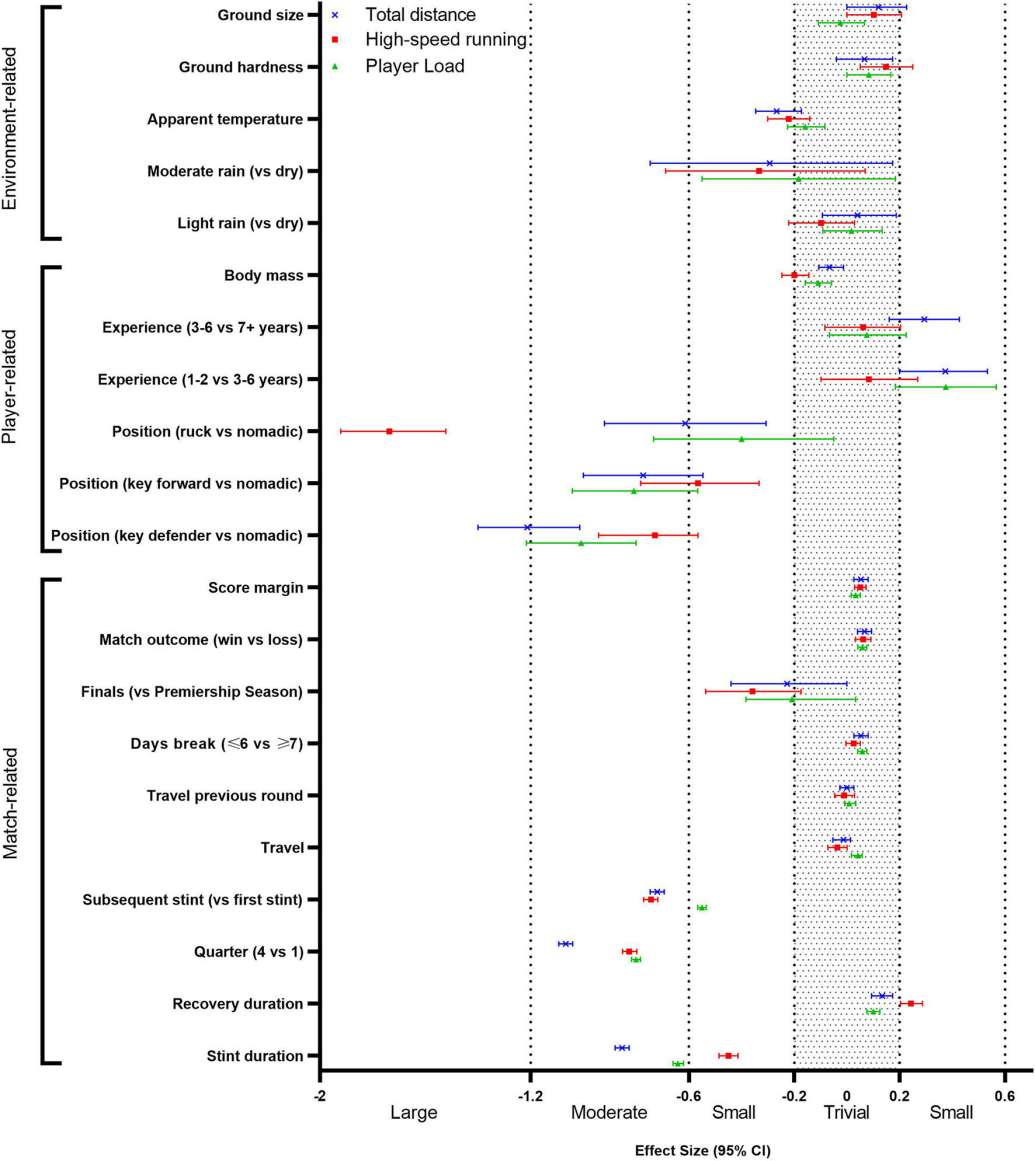
Standardised effects of all independent variables on match activity profile.

The standardised effects of all predictor variables are visualised in Figure 1.

## Discussion

The current study is the largest of its kind to evaluate the effects of a range of match-related, player-related and environment-related factors on match activity profile across an entire league in any sport. The factors associated with lower relative match activity profile were longer stint duration, progression in the stage of the match (quarters) and the season (Finals Series), subsequent stints, playing in a key position, higher body mass, moderate levels of rainfall and higher apparent temperatures. On the other hand, longer recovery duration between stints was associated with increases in relative high-speed running while lower professional experience was associated with higher relative total distance. No substantial effects existed for other variables including travel, days break, match outcome, score margin, ground hardness, and ground size.

An increase in stint duration (+2SD) was associated with small to moderate reductions in all the relative activity profile metrics. This outcome is in agreement with the findings of previous studies in Australian football where the effect of stint duration was evaluated as changes in relative activity profile for various stint duration bins of 2-min intervals (Montgomery and Wisbey, 2016), for increases in stint duration with time as a continuous variable (similar to the current study) (Dillon et al., 2018), or indirectly for a greater number of individual player rotations (Mooney M. et al., 2013; Ryan et al., 2017). In the absence of direct measures for fatigue during match-play due to practical reasons, these findings provide an indication of a gradual increase in fatigue throughout a stint, resulting in lower relative physical output as each on-field stint stretches longer. On the other hand, recovery duration between stints in each quarter had a small positive effect on relative high-speed running and trivial effects on relative total distance and Player Load^TM^. In recent times, Australian football coaches have increasingly used interchange (rotation) as a tactical measure to minimise the negative effects of fatigue on players by reducing stint duration and increasing the passive rest periods between stints. The average number of interchanges for each team in AFL matches increased from <30 per match in 2003 to 133 per match in 2013 (Orchard et al., 2012; Montgomery and Wisbey, 2016). An interchange cap rule was introduced in 2014, limiting the number of interchanges to 120 per match for each team. The interchange cap was later further reduced to 90 per match in 2016, increasing the need to better understand the effects of stint duration and recovery duration to use the limited available interchanges more effectively.

There were small reductions in relative activity profile from one quarter to the next and moderate reductions when comparing the first quarter to the fourth. The magnitude of reduction in relative total distance from the first quarter to the fourth quarter in the current study (8%) was similar to the previously reported reductions of 8–12% (Coutts et al., 2010; Hiscock et al., 2012; Mooney M. et al., 2013). In another study a comparable reduction of ~6% could be inferred from the figures, which was not considered substantial (Aughey, 2010). However, the between-player differences were obtained from the pool of players for the purpose of standardisation, which was slightly different to the position-specific approach taken in the current study and can explain the differences in the interpretation of results. Increasing levels of peripheral and central fatigue throughout the match is the likely reason for the consistent decline in activity profile as the match progresses (Noakes, 2000; Bangsbo et al., 2007; Garrett et al., 2019). The rate of decline for high-speed running was slightly lower in the third quarter following the 20-min half-time break (6.7%) compared to the decline rates in the second and fourth quarters following the 6-min quarter breaks (9.3% and 8.8%). However, such differences of <2.6% are negligible considering the smallest important change of 5.5% for high-speed running. This finding along with the relatively stable decline rates for the other measures of activity profile provide preliminary indications of the limited value of the extended half-time rest period for the recovery of players.

Travelling for the current match or in the previous round did not have any substantial effects on match activity profile. The limited existing evidence on the effects of travel on match activity profile is contradictory in Australian football, where negative effects (Ryan et al., 2017) and no substantial effects (Hiscock et al., 2012) have been reported. Similar inconsistencies in findings on the effects of travel also exist between the studies conducted in other football codes (Lago et al., 2010; Castellano et al., 2011; Kempton and Coutts, 2016; Mitchell et al., 2017; Zhou et al., 2019; Oliva-Lozano et al., 2020). Our findings along with the findings of the only other league-wide study (conducted in the Chinese soccer league) (Zhou et al., 2019) are indicative of the trivial effects of travel on match activity profile on average across a league, or in other words for a typical club. While the negative effects of playing away from home on match outcome is well-established (Pollard, 2006; Lazarus et al., 2017), it has been proposed that tactical, strategic, mental, and officiating factors are responsible, rather than the physical and technical performance of players (Lazarus et al., 2017; Lo et al., 2019). However, we cannot rule out the possibility of between-club differences in the effects of travel, which can explain the observed disagreements in the literature. A closer examination of the random effect (for club identity) across a league and over multiple seasons is required to establish whether travel affects certain clubs substantially more than others. In addition, individual differences between players in the effects of travel may also exist, which can further inform individualised player management practices.

The number of days break between matches did not have a substantial effect on match activity profile, which was in agreement with previous findings. No substantial differences in activity profile has been found between short (6 days) and standard (≥7 days) breaks (Ryan et al., 2017) or between matches of 6, 7, and 8 days break (Hiscock et al., 2012) in Australian football. A higher relative total distance was reported for matches with 12 days break compared to the 6–8 days breaks; however, only two matches were played with this long interval, limiting the generalisability of this finding (Hiscock et al., 2012). The trivial effects of days break (within the established competition scheduling confines) also extend to match outcome in Australian football and rugby (Robertson and Joyce, 2015; Lazarus et al., 2017). Musculoskeletal, neuromuscular, perceptual, and hormonal markers of recovery in Australian footballers return to baseline typically within 2–4 days following a match (Cormack et al., 2008; Gallo et al., 2017; Esmaeili et al., 2018b). The recovery of players is further enhanced through training periodisation and modification strategies implemented by club staff (Aughey et al., 2016; Gallo et al., 2017), leading to the trivial effects of days break on match activity profile. The match turnaround of 6 days, which is generally labelled as short, still provides sufficient opportunity for players to recover for the following match, such that they can reproduce a similar locomotion.

Match outcome and score margin also had trivial effects on match activity profile. These results were in contrast with the findings of two studies conducted at the same Australian football club 4 years apart. Measures of relative activity profile were typically lower during matches (Ryan et al., 2017) and quarters (Sullivan et al., 2014) won, with the exception of higher relative total distance during wins in one study (Ryan et al., 2017). Larger score margins were also associated with a lower activity profile (Sullivan et al., 2014). The differences between the findings on the effects of match outcome and score margin can potentially be due to differences in team game style and tactics (Gronow et al., 2014; Sullivan et al., 2014). While the effects for match outcome and score margin were trivial for a typical club across the AFL, deviations from the mean effect may exist for certain clubs as discussed earlier. In addition, the activity profile varies at different phases of the match, with offence and defence involving higher relative total distance and high-speed running compared to other phases of play including contested play (ball in dispute), stoppages, set shots and goal resets (Rennie et al., 2020). Hence, the possibility of a dynamic two-way relationship between the rolling match outcome status/score margin and activity profile throughout the match should also be considered, which was not possible in the current study. Such complex interactions at different phases of the match in conjunction with the game style of the teams across the league require further investigation.

The relative physical output was lower during the Finals Series compared to the Premiership Season. This finding was in contrast to the evidence from a decade ago (Aughey, 2011b), where relative total distance and relative high-speed running were 11% and 9% higher during the Finals Series compared to the Premiership Season. Two possible reasons can explain the differences in findings. First, the previous study had a limited sample size of eight nomadic players from one AFL club, and data from three Premiership Season matches and three Finals Series matches against the same opponents were analysed (Aughey, 2011b). While this approach allowed for controlling the effects of player position and opposition quality, the small sample size may limit the generalisability of the findings to other clubs. Second, and more importantly, Australian football has tactically evolved from possession style to repossession style during the time-span between the two studies (Woods et al., 2017). Repossession style of football is characterised by a decline in uncontested possessions and a concurrent increase in contested possessions, signifying a greater level of congestion and more stoppages (Woods et al., 2017). These changes negatively affect the relative activity profile, as with greater congestion, players have fewer opportunities to move around the field and at the same time the ball is out of play for longer periods (Ryan et al., 2017). Speculatively, the tendency toward quicker repossession of the ball is even more pronounced during matches of greater importance (finals), resulting in higher levels of congestion and more stoppages, which can explain the observed reductions in relative activity profile during the Finals Series compared to the Premiership Season. Comparisons of the raw outputs at the two season phases for the teams that made the finals were consistent with the findings of the model that adjusted for club identity as a random effect.

Position had moderate to large effects, with nomadics having a higher relative activity profile compared to players in key positions. These results were in agreement with the previously reported positional differences in activity profile (Wisbey et al., 2010; Hiscock et al., 2012; Coutts et al., 2015; Ryan et al., 2017; Dillon et al., 2018). Nomadic players are required to cover larger areas of the field due to their tactical roles within both offensive and defensive phases of play, while key position players have more tactical constraints and less territory to cover (Mooney et al., 2011; Gronow et al., 2014). An unknown proportion of the apparent differences in activity profile between positions has been attributed to the positional differences in the number of interchanges (Wisbey et al., 2010; Coutts et al., 2015). Although nomadic players typically have higher aerobic capacities, they tend to rotate more often and as a result, have typically shorter stint durations and experience more passive recovery during the match compared to players in key positions, which allows them to better fulfil their tactical roles and achieve a higher relative activity profile (Wisbey et al., 2010; Mooney et al., 2011; Coutts et al., 2015). However, it should be highlighted that the estimated values in the current study reflect the pure effects of position, as the effects of other variables including stint duration and recovery duration (but not aerobic capacity) have been adjusted for in the model.

The relative total distance and player Load™ of less experienced players were generally higher than their more experienced counterparts, whereas the differences were trivial for relative high-speed running. These findings were partially in agreement with a study conducted with one AFL club, where less experienced players had higher relative total distance and high-speed running compared to more experienced players (Hiscock et al., 2012). A possible reason for the observed effects of experience on relative total distance is that more experienced players are more skilful at reading the game and they tend to use their energy more efficiently, while less experienced players try to compensate for their lack of experience with greater athleticism (Mooney et al., 2011; Gastin et al., 2013). This theory is supported by the greater technical involvement of more experienced players despite their lower physical output (Gastin et al., 2013). The trivial effects of experience on relative high-speed running in the current study and the differences with the previous findings (Hiscock et al., 2012) could be due to the high tactical and situational dependence of these efforts with large match-to-match variability, which is further compounded by the lower reliability of GPS units in measuring high-speed movements (Kempton et al., 2015; Scott et al., 2016).

Higher body mass was associated with lower relative high-speed running and trivial changes in relative total distance and Player Load^TM^. Assuming a stable proportion of fat-free soft tissue mass, higher body mass translates into higher strength and power (Bilsborough et al., 2015), and as seen in the current study, at the expense of high-speed running capacity where every 1% increase in body mass was associated with ~1% reduction in relative high-speed running. While AFL players have consistently been getting taller and heavier since the official records started in 1913 (Norton et al., 1999), this trend appears to have stabilised over the past decade (Supplementary Material), which coincided with the emergence of a repossession style of football (Woods et al., 2017). Development of game plans with a focus on full ground zones in recent times (Woods et al., 2017) has likely placed more emphasis on the quick movement of players around the ground. Coaches are indeed aware of the trade-off between player body mass and mobility as shown in the current study, resulting in the current dogma in Australian football suggesting that “lighter is better” (Lazarus et al., 2017). While heavier players tend to be more experienced (Bilsborough et al., 2017) and play in key positions (Pyne et al., 2006), the estimated effects of body mass in the current study are adjusted for these confounding variables. It should be considered that the current study evaluated the effects of between-player differences in body mass from a single measurement and without consideration of body composition. Future studies should investigate the effects of within-player changes in fat-free soft tissue mass on physical capacity and match activity profile, in order to further our understanding of physical preparation strategies in modern-day football.

Moderate levels of rainfall and higher apparent temperatures had small negative effects on relative total distance and high-speed running, while ground hardness and ground size had trivial effects on match activity profile. Wet conditions in Australian football have anecdotally been associated with more skill execution errors, less effective possession strategies, and tactical adjustments that lead to more congested and slower matches (Anderson et al., 2018). These tactical adjustments are reflected in the lower number of marking opportunities and set shots as well as increases in the number of throw-ins during wet matches (Appleby and Dawson, 2002; Anderson et al., 2018). The increased levels of congestion and higher number of stoppages associated with wet matches reduce the opportunity for players to move around the ground and increase the out-of-play time (Ryan et al., 2017), which can explain the negative effects of moderate rainfall on relative total distance and high-speed running. The negative effects of increases in apparent temperature on match activity profile are likely due to the pacing strategies moderated by the central nervous system aiming to keep the core temperature below 40°C and prevent exertional heat illness (Armstrong et al., 2007). The negative effects of heat on relative total distance has been demonstrated in Australian football, while relative high-speed running remained unaffected by heat (Duffield et al., 2009; Aughey et al., 2014). It was argued that players tend to reduce their low-speed activity in warmer conditions in order to preserve their high-speed running capacity for more important passages of play (Duffield et al., 2009; Aughey et al., 2014). The contrasting results of the current study in regards to high-speed running can be attributed to the increase in the speed and intensity of Australian football matches in recent times (internal AFL reports), continuing the trend from the past few decades (Norton et al., 1999). The increase in the speed and intensity of matches along with the described recent tactical evolution of Australian football (Woods et al., 2017) could have rendered the pacing strategy of merely reducing low-speed activities insufficient to counter the heat stress in warmer conditions.

There were several limitations associated with the current study. A number of factors are known or suspected to affect match activity profile, which were not included in the analysis. Physical capacity, number of stoppages, team tactics, between-team interactions of game-styles, periodisation and recovery status, shoe-surface traction, training load and detraining following injuries are a few examples (Sterzing et al., 2009; Mooney M. G. et al., 2013; Greenham et al., 2017; Ryan et al., 2017, 2018; Esmaeili et al., 2018a; Rennie et al., 2020). Inclusion of these variables in the analysis can potentially improve the accuracy of the estimated effects. A further limitation of this study was that player position was determined as a fixed value for the entire season, while in reality the tactical role and position of players may change from one match to another or indeed at different phases within a match. It should be also noted that the estimated effects are averages across the AFL and all findings may not necessarily apply to every club in the competition. Generalisation of the findings to lower tier competitions, other sports and female athletes should be done with caution.

## Conclusion

A multitude of factors affect player locomotion in Australian football and should be considered collectively when interpreting match activity profile data. The absolute effects of these factors were typically larger on relative high-speed running compared to the relative total distance and Player Load^TM^, while the standardised effects were relatively similar, owing to the greater between-player differences in high-speed running. Player position and stage of the match (quarter) had the largest effects on activity profile while stint duration, recovery duration, professional experience, body mass, stage of the season and weather conditions also had substantial effects.

## Data Availability Statement

The copyright and confidentiality agreements between the authors and the Australian Football League prohibits the authors from making the data available to third parties. There is also the risk of individual players being re-identified by cross matching the dataset with publicly available information.

## Ethics Statement

The studies involving human participants were reviewed and approved by Victoria University Human Research Ethics Committee. Written informed consent for participation was not required for this study in accordance with the national legislation and the institutional requirements.

## Author Contributions

AE, PC, and RA conceived and designed the study, interpreted the results, edited, and critically revised the manuscript and approved the final version. AE analysed the data and drafted the manuscript and prepared the tables. All authors contributed to the article and approved the submitted version.

## Conflict of Interest

The authors declare that the research was conducted in the absence of any commercial or financial relationships that could be construed as a potential conflict of interest.

## References

[B1] Anderson D. Breed R. Spittle M. Larkin P. (2018). Factors affecting set shot goal-kicking performance in the Australian football league. Percept. Motor Skills 125, 817–833. 10.1177/0031512518781265 29886806

[B2] Appleby B. Dawson B. (2002). Video analysis of selected game activities in Australian rules football. J. Sci. Med. Sport 5, 129–142. 10.1016/S1440-2440(02)80034-2 12188085

[B3] Armstrong L. E. Casa D. J. Millard-Stafford M. Moran D. S. Pyne S. W. Roberts W. O. (2007). American college of sports medicine position stand. Exertional heat illness during training and competition. Med. Sci. Sports Exerc. 39, 556–572. 10.1249/MSS.0b013e31802fa199 17473783

[B4] Aughey R. J. (2010). Australian football player work rate: evidence of fatigue and pacing? Int. J. Sports Physiol. Perform. 5, 394–405. 10.1123/ijspp.5.3.394 20861528

[B5] Aughey R. J. (2011a). Applications of GPS technologies to field sports. Int. J. Sports Physiol. Perform. 6, 295–310. 10.1123/ijspp.6.3.295 21911856

[B6] Aughey R. J. (2011b). Increased high-intensity activity in Elite Australian Football finals matches. Int. J. Sports Physiol. Perform. 6, 367–379. 10.1123/ijspp.6.3.367 21911862

[B7] Aughey R. J. Elias G. P. Esmaeili A. Lazarus B. Stewart A. M. (2016). Does the recent internal load and strain on players affect match outcome in elite Australian football? J. Sci. Med. Sport 19, 182–186. 10.1016/j.jsams.2015.02.005 25804423

[B8] Aughey R. J. Goodman C. A. McKenna M. J. (2014). Greater chance of high core temperatures with modified pacing strategy during team sport in the heat. J. Sci. Med. Sport 17, 113–118. 10.1016/j.jsams.2013.02.013 23689104

[B9] Bangsbo J. Iaia F. M. Krustrup P. (2007). Metabolic response and fatigue in soccer. Int. J. Sports Physiol. Perform. 2:111. 10.1123/ijspp.2.2.111 19124899

[B10] Bilsborough J. C. Greenway K. G. Opar D. A. Livingstone S. G. Cordy J. T. Bird S. R.. (2015). Comparison of anthropometry, upper-body strength, and lower-body power characteristics in different levels of Australian football players. J. Strength Conditioning Res. 29, 826–834. 10.1519/JSC.0000000000000682 25226309

[B11] Bilsborough J. C. Kempton T. Greenway K. Cordy J. Coutts A. J. (2017). Longitudinal changes and seasonal variation in body composition in professional Australian football players. Int. J. Sports Physiol. Perform. 12, 10–17. 10.1123/ijspp.2015-0666 27002304

[B12] Castellano J. Blanco-Villaseñor A. Álvarez D. (2011). Contextual variables and time-motion analysis in soccer. Int. J. Sports Med. 32, 415–421. 10.1055/s-0031-1271771 21590641

[B13] Cormack S. J. Newton R. U. McGuigan M. R. (2008). Neuromuscular and endocrine responses of elite players to an Australian rules football match. Int. J. Sports Physiol. Perform. 3, 359–374. 10.1123/ijspp.3.3.359 19211947

[B14] Coutts A. J. Kempton T. Sullivan C. Bilsborough J. Cordy J. Rampinini E. (2015). Metabolic power and energetic costs of professional Australian Football match-play. J. Sci. Med. Sport 18, 219–224. 10.1016/j.jsams.2014.02.003 24589369

[B15] Coutts A. J. Quinn J. Hocking J. Castagna C. Rampinini E. (2010). Match running performance in elite Australian Rules Football. J. Sci. Med. Sport 13, 543–548. 10.1016/j.jsams.2009.09.004 19853508

[B16] Dillon P. A. Kempton T. Ryan S. Hocking J. Coutts A. J. (2018). Interchange rotation factors and player characteristics influence physical and technical performance in professional Australian Rules football. J. Sci. Med. Sport 21, 317–321. 10.1016/j.jsams.2017.06.008 28629667

[B17] Duffield R. Coutts A. J. Quinn J. (2009). Core temperature responses and match running performance during intermittent-sprint exercise competition in warm conditions. J. Strength Conditioning Res. 23, 1238–1244. 10.1519/JSC.0b013e318194e0b1 19568033

[B18] Esmaeili A. Hopkins W. G. Stewart A. M. Elias G. P. Lazarus B. H. Aughey R. J. (2018a). The individual and combined effects of multiple factors on the risk of soft tissue non-contact injuries in elite team sport athletes. Front. Physiol. 9:1280. 10.3389/fphys.2018.01280 30333756 PMC6176657

[B19] Esmaeili A. Stewart A. M. Hopkins W. G. Elias G. P. Lazarus B. H. Rowell A. E.. (2018b). Normal variability of weekly musculoskeletal screening scores and the influence of training load across an Australian Football League season. Front. Physiol. 9:144. 10.3389/fphys.2018.00144 29535643 PMC5835227

[B20] Gallo T. F. Cormack S. J. Gabbett T. J. Lorenzen C. H. (2017). Self-reported wellness profiles of professional Australian football players during the competition phase of the season. J. Strength Condition. Res. 31, 495–502. 10.1519/JSC.0000000000001515 27243912

[B21] Garrett J. M. Gunn R. Eston R. G. Jakeman J. Burgess D. J. Norton K. (2019). The effects of fatigue on the running profile of elite team sport athletes. A systematic review and meta-analysis. J. Sports Med. Phys. Fitness 59, 1328–1338. 10.23736/S0022-4707.19.09356-3 30758168

[B22] Gastin P. B. Fahrner B. Meyer D. Robinson D. Cook J. L. (2013). Influence of physical fitness, age, experience, and weekly training load on match performance in Elite Australian Football. J. Strength Conditioning Res. 27, 1272–1279. 10.1519/JSC.0b013e318267925f 22820206

[B23] Greenham G. Hewitt A. Norton K. (2017). A pilot study to measure game style within Australian football. Int. J. Perform. Anal. Sport 17, 576–585. 10.1080/24748668.2017.1372163

[B24] Gronow D. Dawson B. Heasman J. Rogalski B. Peeling P. (2014). Team movement patterns with and without ball possession in Australian Football League players. Int. J. Perform. Anal. Sport 14, 635–651. 10.1080/24748668.2014.11868749

[B25] Hiscock D. Dawson B. Heasman J. Peeling P. (2012). Game movements and player performance in the Australian Football League. Int. J. Perform. Anal. Sport 12, 531–545. 10.1080/24748668.2012.11868617

[B26] Hopkins W. G. Marshall S. Batterham A. Hanin J. (2009). Progressive statistics for studies in sports medicine and exercise science. Med. Sci. Sports Exercise 41, 3–13. 10.1249/MSS.0b013e31818cb278 19092709

[B27] Kempton T. Coutts A. J. (2016). Factors affecting exercise intensity in professional rugby league match-play. J. Sci. Med. Sport 19, 504–508. 10.1016/j.jsams.2015.06.008 26117160

[B28] Kempton T. Sullivan C. Bilsborough J. C. Cordy J. Coutts A. J. (2015). Match-to-match variation in physical activity and technical skill measures in professional Australian Football. J. Sci. Med. Sport 18, 109–113. 10.1016/j.jsams.2013.12.006 24444753

[B29] Lago C. Casais L. Dominguez E. Sampaio J. (2010). The effects of situational variables on distance covered at various speeds in elite soccer. Eur. J. Sport Sci. 10, 103–109. 10.1080/17461390903273994

[B30] Lazarus B. H. Hopkins W. G. Stewart A. M. Aughey R. J. (2017). Factors affecting match outcome in elite Australian football: a 14-year analysis. Int. J. Sports Physiol. Perform. 13, 140–144. 10.1123/ijspp.2016-0450 28488906

[B31] Lo M. Aughey R. J. Hopkins W. G. Gill N. Stewart A. M. (2019). The longest journeys in Super Rugby: 11 years of travel and performance indicators. J. Sports Sci. 37, 2045–2050. 10.1080/02640414.2019.1618533 31109247

[B32] Mitchell J. A. Pumpa K. L. Pyne D. B. (2017). Responses of lower-body power and match running demands following long-haul travel in international rugby sevens players. J. Strength Conditioning Res. 31, 686–695. 10.1519/JSC.0000000000001526 27359207

[B33] Montgomery P. G. Wisbey B. (2016). The effect of interchange rotation period and number on Australian football running performance. J. Strength Conditioning Res. 30, 1890–1897. 10.1519/JSC.0000000000000597 27328273

[B34] Mooney M. Cormack S. O'Brien B. Coutts A. J. (2013). Do physical capacity and interchange rest periods influence match exercise-intensity profile in australian football? Int J Sports Physiol Perform. 8:165. 10.1123/ijspp.8.2.165 23428488

[B35] Mooney M. O'Brien B. Cormack S. Coutts A. Berry J. Young W. (2011). The relationship between physical capacity and match performance in elite Australian football: a mediation approach. J. Sci. Med. Sport 14, 447–452. 10.1016/j.jsams.2011.03.010 21530392

[B36] Mooney M. G. Cormack S. O'Brien B. J. Morgan W. M. McGuigan M. (2013). Impact of neuromuscular fatigue on match exercise intensity and performance in elite Australian football. J. Strength Conditioning Res. 27, 166–173. 10.1519/JSC.0b013e3182514683 22395264

[B37] Murray N. B. Gabbett T. J. Chamari K. (2014). Effect of different between-match recovery times on the activity profiles and injury rates of national rugby league players. J. Strength Conditioning Res. 28, 3476–3483. 10.1519/JSC.0000000000000603 24983851

[B38] Noakes T. D. (2000). Physiological models to understand exercise fatigue and the adaptations that predict or enhance athletic performance. Scand. J. Med. Sci. Sports 10, 123–145. 10.1034/j.1600-0838.2000.010003123.x 10843507

[B39] Norton K. I. Craig N. Olds T. (1999). The evolution of Australian football. J. Sci. Med. Sport 2, 389–404. 10.1016/S1440-2440(99)80011-5 10710016

[B40] Oliva-Lozano J. M. Rojas-Valverde D. Gómez-Carmona C. D. Fortes V. Pino-Ortega J. (2020). Impact of contextual variables on the representative external load profile of spanish professional soccer match-play: a full season study. Eur. J. Sport Sci. 12:1–22. 10.1080/17461391.2020.1751305 32233969

[B41] Orchard J. W. Driscoll T. Seward H. Orchard J. J. (2012). Relationship between interchange usage and risk of hamstring injuries in the Australian Football League. J. Sci. Med. Sport 15, 201–206. 10.1016/j.jsams.2011.11.250 22197066

[B42] Pollard R. (2006). Worldwide regional variations in home advantage in association football. J. Sports Sci. 24, 231–240. 10.1080/02640410500141836 16368633

[B43] Pyne D. B. Gardner A. S. Sheehan K. Hopkins W. G. (2006). Positional differences in fitness and anthropometric characteristics in Australian football. J. Sci. Med. Sport 9, 143–150. 10.1016/j.jsams.2005.10.001 16580878

[B44] Ramírez-López C. Till K. Boyd A. Bennet M. Piscione J. Bradley S.. (2020). Coopetition: cooperation among competitors to enhance applied research and drive innovation in elite sport. Br. J. Sports Med. bjsports-2020–102901. 10.1136/bjsports-2020-102901 32747407

[B45] Rennie M. J. Kelly S. J. Bush S. Spurrs R. W. Austin D. J. Watsford M. L. (2020). Phases of match-play in professional Australian Football: distribution of physical and technical performance. J. Sports Sci. 38, 1682–1689. 10.1080/02640414.2020.1754726 32342727

[B46] Robertson S. J. Joyce D. G. (2015). Informing in-season tactical periodisation in team sport: development of a match difficulty index for Super Rugby. J. Sports Sci. 33, 99–107. 10.1080/02640414.2014.925572 24977714

[B47] Ryan S. Coutts A. J. Hocking J. Dillon P. A. Whitty A. Kempton T. (2018). Physical preparation factors that influence technical and physical match performance in professional Australian football. Int. J. Sports Physiol. Perform. 13:1021. 10.1123/ijspp.2017-0640 29466065

[B48] Ryan S. Coutts A. J. Hocking J. Kempton T. (2017). Factors affecting match running performance in professional Australian football. Int. J. Sports Physiol. Perform. 12, 1199–1204. 10.1123/ijspp.2016-0586 28182505

[B49] Scott M. T. Scott T. J. Kelly V. G. (2016). The validity and reliability of global positioning systems in team sport: a brief review. J. Strength Conditioning Res. 30, 1470–1490. 10.1519/JSC.0000000000001221 26439776

[B50] Steadman R. G. (1994). Norms of apparent temperature in Australia. Australian Meteorol. Magazine 43, 1–16.

[B51] Sterzing T. Müller C. Hennig E. M. Milani T. L. (2009). Actual and perceived running performance in soccer shoes: a series of eight studies. Footwear Sci. 1, 5–17. 10.1080/19424280902915350

[B52] Sullivan C. Bilsborough J. C. Cianciosi M. Hocking J. Cordy J. Coutts A. J. (2014). Match score affects activity profile and skill performance in professional Australian Football players. J. Sci. Med. Sport 17, 326–331. 10.1016/j.jsams.2013.05.001 23770325

[B53] Wisbey B. Montgomery P. G. Pyne D. B. Rattray B. (2010). Quantifying movement demands of AFL football using GPS tracking. J. Sci. Med. Sport 13, 531–536. 10.1016/j.jsams.2009.09.002 19897414

[B54] Woods C. T. Robertson S. Collier N. F. (2017). Evolution of game-play in the Australian Football League from 2001 to 2015. J. Sports Sci. 35, 1879–1887. 10.1080/02640414.2016.1240879 27732158

[B55] Zhou C. Hopkins W. G. Mao W. Calvo A. L. Liu H. (2019). Match performance of soccer teams in the chinese super League—effects of situational and environmental factors. Int. J. Environ. Res. Public Health 16:4238. 10.3390/ijerph16214238 31683754 PMC6862007

