# Supplementary figures and images for: A League-Wide Evaluation of Factors Influencing Match Activity Profile in Elite Australian Football
Source: Front Sports Act Living. 2020 Nov 6;2:579264. doi: 10.3389/fspor.2020.579264 (PMC7739775; doi:10.3389/fspor.2020.579264)

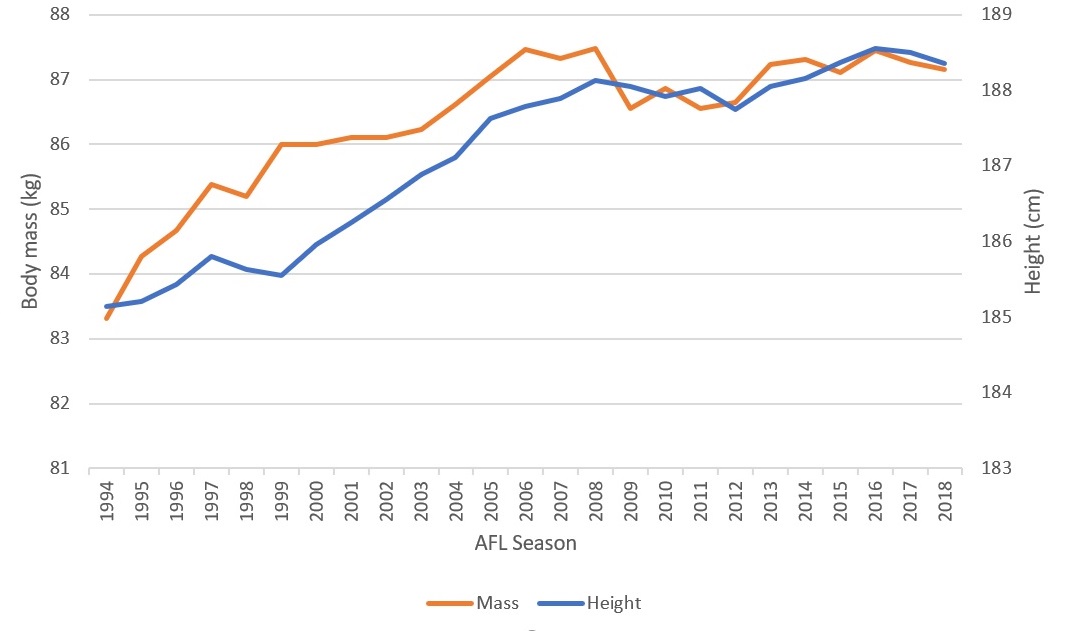

Supplement: Supplementary file 1 [file Image_1.JPEG]
